# Late-match card-event rates and score-state associations in Barcelona La Liga matches featuring Lionel Messi: a retrospective analysis of StatsBomb open event data

**DOI:** 10.3389/fpsyg.2026.1992963

**Published:** 2026-10-28

**Authors:** Tianran Gong, Yeqin Zhang

**Affiliations:** China Football College, Beijing Sport University, Beijing, China

**Keywords:** card events, match period, retrospective study, score state, soccer refereeing, StatsBomb

## Abstract

**Objective:**

We examined whether card-event rates were higher in the final 15 min of regulation time and second-half stoppage time than in earlier periods (H1), and whether home-trailing status at the start of a time window was associated with subsequent home- and away-team card-event rates (H2). A secondary, exploratory analysis compared temporal associations before and from the 2018/19 season onward (H3), using an era proxy rather than actual video assistant referee (VAR) exposure.

**Methods:**

This retrospective observational study included 519 FC Barcelona La Liga matches from 2004/05 to 2020/21 in StatsBomb Open Data in which Lionel Messi had a recorded playing position and the required files were available. H1 and H3 used three periods (0–75 min, 75–90 min, and second-half stoppage time), whereas H2 used six 15-min regulation-time windows and a second-half stoppage-time window. Outcomes comprised Yellow Card, Second Yellow, and Red Card events. Poisson regression used exposure-time offsets and match-clustered robust standard errors; the primary H1 model adjusted for recorded foul intensity and other covariates.

**Results:**

Across 3,114 team-by-period observations, adjusted total card-event rates were higher at 75–90 min (incidence rate ratio [IRR] = 1.487, 95% CI 1.353–1.634) and in second-half stoppage time (IRR = 1.766, 95% CI 1.480–2.108), both *p* < 0.001. For Foul Committed-linked cards, the corresponding IRRs were 1.324 (95% CI 1.195–1.467) and 0.967 (95% CI 0.776–1.203). The Bad Behaviour share increased descriptively from 16.11% to 43.09%. Home-trailing status at window onset was not significantly associated with subsequent away-team card-event rates (IRR = 0.981, 95% CI 0.858–1.121). Era-by-period interactions provided no clear evidence of change.

**Conclusion:**

Late-match total card-event rates were higher conditional on recorded foul intensity, and the stoppage-time card composition shifted toward Bad Behaviour. The analyses did not formally establish different temporal associations by event source. These patterns do not demonstrate decision inaccuracy or referee bias.

## 1 Introduction

Soccer referees make decisions under crowd noise, outcome pressure, and time constraints. Experimental evidence indicates that crowd noise can influence referee judgments (Nevill et al., 2002), while observational studies of added time and matches played behind closed doors suggest associations between social pressure and some officiating outcomes (Garicano et al., 2005; Pettersson-Lidbom and Priks, 2010; Bryson et al., 2021; Reade et al., 2022). Video assistant refereeing (VAR) was introduced in La Liga in the 2018/19 season (LaLiga, 2018), although findings across leagues differ regarding its relationship with home advantage and disciplinary outcomes (Dufner et al., 2023; Gasparetto and Loktionov, 2023; Işın and Yi, 2024). Temporal patterns in card events and their associations with recorded foul intensity and score state warrant further event-level investigation.

The closing stages of a match may involve time pressure, score uncertainty, and player protest. Cognitive and physical factors in referee decision-making have been investigated experimentally and observationally (Samuel et al., 2018; Castillo-Rodríguez et al., 2023; Wang et al., 2024; Zhang et al., 2024), but these mechanisms cannot be inferred directly from associations between match time and card events. We therefore distinguished three analytical questions: card-event rates across three match periods, associations with score state at the onset of seven time windows, and exploratory differences in temporal associations across eras.

The present study focused on Barcelona La Liga matches featuring Lionel Messi in StatsBomb (2026). This restricted, availability-based sampling frame maintained a consistent club and participation criterion despite incomplete league-wide open-data coverage; it does not represent La Liga as a whole. H1 and H2 were the primary hypotheses. H1 tested whether card-event rates were higher at 75–90 min and in second-half stoppage time than at 0–75 min. H2 examined whether home-trailing status at window onset was associated with subsequent home- and away-team card-event rates, with the directional expectation of a higher away-team rate. H3 was a secondary, exploratory comparison of temporal associations before versus from 2018/19 onward. The cut-off corresponds to the introduction of VAR in LaLiga (2018), but the era comparison does not evaluate actual VAR interventions.

## 2 Materials and methods

### 2.1 Study design and data source

This was a retrospective, non-interventional observational study reported with reference to the Strengthening the Reporting of Observational Studies in Epidemiology (STROBE) statement (von Elm et al., 2007). Data were obtained from the official StatsBomb Open Data repository (StatsBomb, 2026). In its competition directory, competition_id = 11 identifies the Spanish first division (La Liga) for data retrieval. We read match, lineup, and event files for the 2004/05 through 2020/21 seasons. A non-null position record for Lionel Messi in the lineup file was used as the operational criterion for actual participation.

The final sampling frame comprised 519 Barcelona La Liga matches in which Messi met this participation criterion and the required files were available. Restricting the sample to one club and matches featuring one player provides an explicit, reproducible scope, but introduces club-, player-, and data-coverage selection. The findings should not be generalized to average card-event patterns across La Liga. Each match contributed two teams across three analysis periods, yielding 3,114 team-by-period observations for H1 and H3.

All data were publicly available match events. The referee field was used only for completeness checks and a sensitivity analysis restricted to matches with non-missing referee information. This field was missing for 105 matches. Referee identity was not included in the primary or sensitivity models, and between-referee heterogeneity was not modeled.

### 2.2 Inclusion and exclusion criteria

Matches were eligible when (1) match, lineup, and event files were available; (2) Messi had a recorded playing position; and (3) the final score could be reconstructed from Shot events labeled Goal and Own Goal Against. Reconstructed final scores matched the match metadata for all 519 matches; no event file was excluded because of a score discrepancy.

### 2.3 Variable definitions and event-stream processing

Match periods. Period-specific timestamps, rather than rounded minute values, were used. For H1 and H3, first-half timestamps from 0:00 up to but not including 45:00 and second-half timestamps from 0:00 up to but not including 30:00 were combined as 0–75 min. Second-half timestamps from 30:00 up to but not including 45:00 defined 75–90 min, and timestamps of 45:00 or later defined second-half stoppage time (labeled >90 min). The 75-min cut-off was a fixed operational threshold chosen to identify the final 15 min of regulation time, rather than an empirically optimized split or evidence of a mechanistic change at that minute. First-half stoppage time falls outside these three mutually exclusive periods and was excluded. It would provide a potentially informative comparison, but no descriptive or sensitivity analysis of that period was performed. Second-half stoppage-time exposure was estimated as the timestamp of the final recorded event in period 2 minus 45 min. This is a proxy for duration observable in the event stream, not the official actual stoppage-time duration.

Outcomes. We extracted Yellow Card, Second Yellow, and Red Card sanctions recorded within Foul Committed and Bad Behaviour events. The primary outcome was the total number of card events received by each team within each analysis period. Foul Committed-linked cards were used in an outcome-definition sensitivity analysis, and Bad Behaviour cards in a descriptive source decomposition. Because Bad Behaviour card records do not have a corresponding Foul Committed denominator, we did not calculate a foul-to-card conversion rate. Recorded fouls are influenced by referee recognition and event recording and are not a behavioral gold standard independent of officiating.

Score state. H2 divided regulation time into six 15-min windows (0–15, 15–30, 30–45, 45–60, 60–75, and 75–90 min) and added a second-half stoppage-time window. First-half stoppage time was excluded. The home team’s net score was reconstructed using goals that had occurred before each window began; a negative net score defined home-trailing status at window onset. This status was assigned to the entire subsequent window. Goals within a window did not trigger subdivision or reassignment of score-state exposure. H2 therefore estimates associations between score state at window onset and subsequent window-level card-event rates, not instantaneous rates per minute actually spent trailing. The model included an interaction between away-team role and this score-state indicator.

Era indicator. H3 categorized seasons as pre-2018/19 or 2018/19 onward because VAR was introduced in La Liga in 2018/19 (LaLiga, 2018). The open event files do not identify individual VAR interventions. This indicator may also capture contemporaneous changes in laws, refereeing guidance, team composition, data vintage, and pandemic conditions. H3 was therefore a secondary, exploratory era comparison, not a direct measurement of VAR exposure or an estimate of its causal effect.

Empty-stadium proxy. The open files do not provide match-level attendance. Based on match dates, 46 matches played from 11 June 2020 through 23 May 2021 were coded as an empty-stadium proxy, with the remaining 473 forming the comparison group. This variable was included for adjustment and stratified robustness checks to address potential confounding by pandemic-period spectator conditions. Estimating a crowd effect was not a study objective. The classification was not verified against match-level attendance and overlaps strongly with the era indicator.

*Covariates*. Models included team role, recorded fouls per 15 min within the same analysis period, the empty-stadium indicator, season fixed effects, and team fixed effects as specified below. The official event data do not contain a directly verifiable season-ending league-position variable, so final opponent ranking was not included. Team fixed effects were used to control for stable differences across Barcelona and its opponents.

### 2.4 Statistical analysis

Data processing and estimation were performed in Python 3.12 using NumPy, pandas, and SciPy. Tests were two-sided with alpha = 0.05. Continuous variables are reported as mean ± SD, and count variables as frequencies and proportions. These summaries are descriptive; inference about period differences is based on the regression models with exposure offsets and match-clustered robust standard errors described below.

For H1, Y denotes the card-event count for team i in match m and period *t*, and T denotes the period duration in minutes. The Poisson model with a log link was specified as follows:


$$\begin{array}{c} \log[E(Y_{\mathrm{mit}})]=\log(T_{\mathrm{mt}})+\beta_{0}+\beta_{1}I(75-90\;\min)+\beta_{2}I(>90\;\min)\\ +\beta_{3}F_{\mathrm{mit}}+\gamma^{T}X_{\mathrm{mit}} \end{array}$$

The covariate vector X contains team role, the empty-stadium proxy, season fixed effects, and team fixed effects, with coefficient vector *γ*; F is the number of recorded fouls per 15 min in the same period. The reference period is 0–75 min, and the natural log of period duration is an offset with its coefficient fixed at 1. Sandwich robust standard errors were clustered by match_id. *β* denotes a regression coefficient on the log event-rate scale, and IRR = exp.(*β*) is the rate ratio conditional on the included covariates. Wald 95% confidence intervals were obtained by exponentiating *β* ± 1.96 × robust SE; two-sided Wald *p* values test the corresponding coefficient against zero, equivalently IRR = 1. For the primary model, Pearson chi-square/*d*f = 0.896 and the method-of-moments negative-binomial dispersion estimate was 0, providing no evidence of meaningful overdispersion. Same-period recorded foul intensity may reflect both behavior and officiating/recording processes. The primary model therefore estimates a temporal association conditional on recorded fouls, rather than the overall temporal association without this adjustment. A temporal model omitting recorded foul intensity was not fitted; the overall and foul-conditional temporal associations therefore cannot be compared directly.

For H2, 7,266 team-by-window observations were analyzed using a Poisson model with log(window duration) as an offset. The core predictors were home-trailing status at window onset, team role, and their interaction. The model also included window, season, and team fixed effects, recorded foul intensity in the same window, and the empty-stadium proxy. Standard errors were clustered by match. Linear combinations of coefficients estimated the home- and away-team IRRs comparing windows that began with the home team trailing against windows that began level or leading.

For H3, the analysis returned to the three-period dataset of 3,114 observations. A Poisson model with the same exposure offset and match-clustered robust standard errors included period × era interactions, the era indicator, a linear season trend, team fixed effects, and the remaining H1 covariates. Because the era indicator is perfectly collinear with a full set of season fixed effects, H3 did not include season fixed effects. Table 1 summarizes the analytical units, exposures, outcomes, and model structures for the three questions.

**Table 1:** Analytical framework for the three study questions.

Question and status	Analytical unit and time structure	Outcome and principal exposure	Model and interpretation
H1 Primary	Team × match × three periods *n* = 3,114	All card events 0–75, 75–90, and >90 min	Poisson; log-duration offset; match-clustered robust SE; adjustment for same-period recorded foul intensity, team role, empty-stadium proxy, season and team fixed effects.
H2 Primary	Team × match × seven windows *n* = 7,266	All card events Home-trailing status at window onset and its interaction with team role	Poisson; log-duration offset; match-clustered robust SE; window, season and team fixed effects, recorded foul intensity and empty-stadium proxy. Estimates subsequent window-level conditional associations.
H3 Secondary exploratory	Team × match × three periods *n* = 3,114	All card events Period × 2018/19-or-later era	Poisson; log-duration offset; match-clustered robust SE; linear season trend, team fixed effects and other H1 covariates. Era proxy, not actual VAR exposure.

The outcome-definition sensitivity analysis restricted total cards to Foul Committed-linked cards. Stratified analyses repeated the H1 specification in the spectator-proxy comparison group, empty-stadium-proxy matches, Barcelona home matches, Barcelona away matches, matches with complete referee fields, and seasons from 2012/13 onward. The complete-referee-field analysis restricted the sample to 414 matches with non-missing referee information; referee identity was not added to the model, so this restriction does not adjust for between-referee heterogeneity. All analyses were associational. No independent video-based adjudication of decision correctness was performed, and exploratory window-specific and subgroup comparisons were not adjusted for multiple testing.

## 3 Results

### 3.1 Season-level sample characteristics

The final sample included 519 matches across 17 seasons: 417 before 2018/19 and 102 from 2018/19 onward. Barcelona was the home team in 261 matches and the away team in 258; 46 matches met the empty-stadium proxy definition. The number of included matches varied substantially by season, with only seven matches in 2004/05, reflecting the combination of open-data availability and Messi’s participation rather than complete season sampling (Table 2).

**Table 2:** Season-level characteristics of included matches (*n* = 519).

Season	Matches	Era	Empty-stadium proxy, *n*	Recorded fouls per match	Yellow/s-yellow cards per match	Red cards per match	Second-half stoppage time, min
2004/05	7	Pre-2018/19	0	42.00 ± 4.43	5.71 ± 2.29	0.00 ± 0.00	3.54 ± 2.06
2005/06	17	Pre-2018/19	0	45.29 ± 8.74	4.88 ± 2.39	0.18 ± 0.39	2.93 ± 1.41
2006/07	26	Pre-2018/19	0	38.42 ± 9.44	5.19 ± 2.80	0.35 ± 0.69	3.31 ± 1.56
2007/08	27	Pre-2018/19	0	37.11 ± 6.38	4.93 ± 3.02	0.11 ± 0.32	3.14 ± 1.08
2008/09	31	Pre-2018/19	0	35.35 ± 6.67	5.00 ± 2.62	0.19 ± 0.40	3.20 ± 1.60
2009/10	35	Pre-2018/19	0	36.69 ± 7.99	5.23 ± 2.62	0.17 ± 0.51	2.76 ± 1.16
2010/11	33	Pre-2018/19	0	31.88 ± 9.16	4.94 ± 2.74	0.09 ± 0.29	2.95 ± 1.31
2011/12	37	Pre-2018/19	0	32.97 ± 6.69	5.89 ± 2.89	0.05 ± 0.23	2.95 ± 1.39
2012/13	32	Pre-2018/19	0	30.78 ± 7.74	4.41 ± 2.30	0.06 ± 0.25	2.99 ± 0.80
2013/14	31	Pre-2018/19	0	32.29 ± 7.15	4.52 ± 1.75	0.06 ± 0.25	3.10 ± 0.76
2014/15	38	Pre-2018/19	0	31.11 ± 6.98	4.79 ± 2.95	0.13 ± 0.34	3.00 ± 0.98
2015/16	33	Pre-2018/19	0	29.42 ± 6.81	4.91 ± 2.28	0.15 ± 0.44	2.55 ± 0.98
2016/17	34	Pre-2018/19	0	32.53 ± 7.48	4.94 ± 2.31	0.09 ± 0.29	3.11 ± 1.05
2017/18	36	Pre-2018/19	0	29.00 ± 5.41	4.28 ± 2.04	0.08 ± 0.28	3.22 ± 0.98
2018/19	34	2018/19+	0	29.18 ± 5.35	4.68 ± 2.43	0.09 ± 0.29	3.63 ± 1.20
2019/20	33	2018/19+	11	29.30 ± 5.66	4.61 ± 2.40	0.15 ± 0.51	4.17 ± 1.31
2020/21	35	2018/19+	35	26.77 ± 6.70	3.97 ± 2.20	0.11 ± 0.32	4.02 ± 1.24
Overall	519	—	46	32.57 ± 8.11	4.83 ± 2.51	0.12 ± 0.37	3.20 ± 1.26

Foul and card counts are restricted to the three analysis periods; first-half stoppage-time events are excluded. Yellow-card counts include second-yellow cards. Era grouping compares seasons before 2018/19 with 2018/19 onward and does not represent observed VAR interventions.

### 3.2 Data availability and quality checks

Match metadata, lineups, and event streams were available for all 519 matches, and final scores reconstructed event by event matched the match metadata in every case. The referee field was complete for 414 matches (79.77%). Match-level attendance and actual VAR intervention records were unavailable. The detailed field inventory, proxy definitions, and verification results are provided in Supplementary Table S1.

### 3.3 Recorded foul intensity and card-event rates by match period

Recorded fouls per 15 min were 2.59 ± 1.06, 2.70 ± 1.74, and 2.71 ± 3.54 in the 0–75-min, 75–90-min, and >90-min periods, respectively. Total card-event rates were 0.35 ± 0.27, 0.55 ± 0.76, and 0.75 ± 1.93 cards per 15 min, respectively (Table 3). These summaries describe observed event density. Associations between periods and their uncertainty are presented in the regression models; neither statistical significance nor decision quality can be inferred from means and SDs alone.

**Table 3:** Recorded fouls and card-event rates across the three analysis periods (*n* = 1,038 team observations per period).

Measure	0–75 min	75–90 min	>90 min	Operational definition	Inferential treatment
Mean period duration, min	75.00	15.00	3.20	Fixed or timestamp-estimated exposure	Descriptive only
Foul events per 15 min	2.59 ± 1.06	2.70 ± 1.74	2.71 ± 3.54	Foul committed	Descriptive only
All cards per 15 min	0.35 ± 0.27	0.55 ± 0.76	0.75 ± 1.93	Yellow + second yellow + red	See Table 6
Foul-linked cards per 15 min	0.29 ± 0.24	0.42 ± 0.64	0.42 ± 1.36	Card attached to foul committed	See Table 6
Bad behaviour cards per 15 min	0.06 ± 0.11	0.13 ± 0.36	0.33 ± 1.35	Card attached to bad behaviour	Exploratory decomposition

Exposure was fixed at 75 min for 0–75 min and 15 min for 75–90 min; >90-min exposure was estimated from the final recorded event timestamp in the second half. First-half stoppage time was excluded. Means ± SD are descriptive only; inferential estimates are provided in Table 6.

### 3.4 Associations between score state at window onset and subsequent card-event rates

In H2, home-trailing status always refers to score state at window onset. Across all seven windows, this status was not significantly associated with the home team’s subsequent card-event rate (IRR = 1.082, 95% CI 0.947–1.236, *p* = 0.245). The corresponding away-team association was IRR = 0.981 (95% CI 0.858–1.121, *p* = 0.775), and the home-away effect difference was not significant (interaction IRR = 0.906, *p* = 0.296). The away-team IRRs were 0.835 (*p* = 0.210) at 75–90 min and 0.770 (*p* = 0.288) at >90 min. In the >90-min window, the home-team IRR was 0.589 (95% CI 0.353–0.982, *p* = 0.042), but the home-away interaction was not significant (*p* = 0.493), and this was an exploratory comparison without multiplicity adjustment. These results do not support the directional expectation of more subsequent away-team cards following home-trailing status at window onset (Table 4). They do not estimate associations during the actual time spent trailing.

**Table 4:** Match-clustered robust Poisson models of home-trailing status at window onset and subsequent card-event rates.

Analysis range	Outcome team/effect	*β*	Robust SE	IRR	95% CI	*p*
All seven windows	Home team	0.079	0.068	1.082	0.947–1.236	0.245
All seven windows	Away team	−0.019	0.068	0.981	0.858–1.121	0.775
All seven windows	Home-away effect difference	−0.098	0.094	0.906	0.754–1.090	0.296
75–90 min	Home team	0.159	0.134	1.173	0.901–1.525	0.235
75–90 min	Away team	−0.180	0.144	0.835	0.630–1.107	0.210
75–90 min	Home-away effect difference	−0.339	0.214	0.712	0.469–1.082	0.112
>90 min	Home team	−0.530	0.261	0.589	0.353–0.982	0.042
>90 min	Away team	−0.262	0.246	0.770	0.475–1.247	0.288
>90 min	Home-away effect difference	0.268	0.391	1.308	0.608–2.813	0.493

*β* denotes the Poisson coefficient on the log event-rate scale; IRR = exp(*β*). For home- and away-team estimates obtained by linear combinations, *β* denotes the corresponding combined coefficient and SE its robust standard error. Two-sided Wald *p*-values test whether the relevant coefficient is zero. IRRs compare windows beginning with the home team trailing against windows beginning level or leading within the same analysis range. All models used log(window duration) as an offset. The pooled model adjusted for window, season, team, role, recorded foul intensity, and the empty-stadium proxy. Window-specific models adjusted for season, team, and the remaining covariates. The home-away effect difference is the interaction term and its IRR is the ratio of the away-team to home-team score-state IRRs. Exploratory comparisons were not adjusted for multiple testing.

### 3.5 Decomposition of card-event sources

Among 2,571 card events included in the analysis, 2,068 (80.44%) were attached to Foul Committed events and 503 (19.56%) to Bad Behaviour events (Table 5). The Bad Behaviour share increased descriptively from 16.11% at 0–75 min to 22.77% at 75–90 min and 43.09% at >90 min. Bad Behaviour counts were not modeled directly, and no event-source × period interaction was tested; this decomposition does not establish a statistical difference in temporal associations between the two sources.

**Table 5:** Card-event counts by event source.

Event source	Yellow card	Second yellow	Red card	Total	% of all cards
Foul committed	1,955	59	54	2,068	80.44
Bad behaviour	486	7	10	503	19.56
Total	2,441	66	64	2,571	100.00

Yellow Card, Second Yellow, and Red Card are counted as recorded events. Bad Behaviour cards have no corresponding Foul Committed denominator and therefore must not be incorporated into a foul-to-card conversion rate.

### 3.6 Multivariable analysis of card-event rates

In the adjusted H1 model, total card-event rates were higher at 75–90 min than at 0–75 min (IRR = 1.487, 95% CI 1.353–1.634, *p* < 0.001) and at >90 min (IRR = 1.766, 95% CI 1.480–2.108, *p* < 0.001). For Foul Committed-linked cards, the 75–90-min IRR was 1.324 (95% CI 1.195–1.467, *p* < 0.001), and the >90-min IRR was 0.967 (95% CI 0.776–1.203, *p* = 0.761). A difference in significance between these separate models does not establish a statistical difference between their effects. Together with the descriptive source decomposition, the findings are consistent with a larger Bad Behaviour share in stoppage time, but do not quantify its independent contribution.

Under the same model conditions, away teams had a higher total card-event rate than home teams (IRR = 1.140, 95% CI 1.069–1.215, *p* < 0.001). Each additional recorded foul per 15 min was associated with an IRR of 1.211 (95% CI 1.183–1.240, *p* < 0.001). The empty-stadium proxy main-effect IRR was 0.879 (*p* = 0.505). These are conditional associations and do not establish decision correctness, referee bias, or a crowd effect (Table 6).

**Table 6:** Poisson models for all card events and foul committed-linked card events.

Outcome/variable	*β*	Robust SE	IRR	95% CI	*z*	*p*
All cards: 75–90 vs. 0–75 min	0.397	0.048	1.487	1.353–1.634	8.229	<0.001
All cards: >90 vs. 0–75 min	0.569	0.090	1.766	1.480–2.108	6.304	<0.001
Away vs. home team	0.131	0.033	1.140	1.069–1.215	3.979	<0.001
Recorded foul intensity: +1 foul/15 min	0.192	0.012	1.211	1.183–1.240	16.110	<0.001
Empty-stadium proxy vs. comparison group	−0.129	0.194	0.879	0.601–1.285	−0.666	0.505
Foul-linked cards: 75–90 vs. 0–75 min	0.281	0.052	1.324	1.195–1.467	5.352	<0.001
Foul-linked cards: >90 vs. 0–75 min	−0.034	0.112	0.967	0.776–1.203	−0.305	0.761

*n* = 3,114 observations clustered within 519 matches. The offset was the natural log of period duration in minutes. Models adjusted for team role, recorded fouls per 15 min in the same period, the empty-stadium proxy, season fixed effects, and team fixed effects. *β* is the coefficient on the log event-rate scale, IRR = exp(*β*), and two-sided Wald *p*-values test *β* = 0. The Foul Committed-linked model was an outcome-definition sensitivity analysis. Estimates from a temporal model without adjustment for recorded foul intensity are not provided.

### 3.7 Temporal effects before versus from the 2018/19 season onward

In the era-interaction model, the IRR for 75–90 min × 2018/19-or-later was 1.242 (95% CI 0.980–1.574, *p* = 0.072), and the IRR for >90 min × 2018/19-or-later was 0.815 (95% CI 0.556–1.197, *p* = 0.297). Neither interaction met the conventional 0.05 significance threshold. The era main effect was also non-significant (IRR = 0.994, *p* = 0.947). Thus, the data did not provide sufficient evidence that the late-period associations changed clearly from the 2018/19 season onward.

The H3 model included a linear season trend, team fixed effects, team role, recorded foul intensity, and the empty-stadium proxy. Only 102 matches occurred in 2018/19 or later, and the interaction confidence intervals remain compatible with both increases and decreases. Non-significant interactions therefore do not establish equivalence between eras. Because the era indicator does not identify actual VAR interventions, these results should not be interpreted as evidence that VAR had no effect (Table 7).

**Table 7:** Era-by-period interaction model comparing pre-2018/19 with 2018/19 onward.

Variable	*β*	Robust SE	IRR	95% CI	*p*
2018/19 + vs. pre-2018/19	−0.006	0.084	0.994	0.843–1.173	0.947
75–90 min × 2018/19+	0.217	0.121	1.242	0.980–1.574	0.072
>90 min × 2018/19+	−0.204	0.196	0.815	0.556–1.197	0.297
Away vs. home team	0.130	0.033	1.139	1.068–1.214	<0.001
Recorded foul intensity: +1 foul/15 min	0.189	0.012	1.209	1.181–1.237	<0.001

The dependent variable was the total card-event count, with log(period duration in minutes) as the offset. The model included a linear season trend, team fixed effects, the empty-stadium proxy, team role, and recorded foul intensity, with match-clustered robust standard errors. *β* denotes the coefficient on the log event-rate scale, IRR = exp(*β*), and two-sided Wald *p*-values test *β* = 0. For period × era interactions, an IRR below or above 1 indicates a weaker or stronger relative period association in the later era, respectively. The era indicator is a proxy, not a causal estimate of actual VAR intervention.

### 3.8 Sensitivity and subgroup analyses

Across the spectator-proxy comparison group, empty-stadium-proxy matches, Barcelona home matches, Barcelona away matches, matches with complete referee fields, and seasons from 2012/13 onward, the directions of the 75–90-min and >90-min estimates for total cards were consistent with the primary analysis (Table 8). The 75–90-min IRRs ranged from 1.375 to 1.849, and the >90-min IRRs from 1.547 to 1.886. The empty-stadium subgroup contained only 46 matches and does not establish a crowd effect. The complete-referee-field subgroup is a complete-case restriction and does not adjust for referee identity.

**Table 8:** Sensitivity analyses of temporal effects on total card-event rates.

Analysis sample	Matches	Observations	75–90 vs. 0–75 min, IRR (95% CI)	*p*	>90 vs. 0–75 min, IRR (95% CI)	*p*
Full sample	519	3,114	1.487 (1.353–1.634)	<0.001	1.766 (1.480–2.108)	<0.001
Spectator proxy	473	2,838	1.462 (1.325–1.613)	<0.001	1.769 (1.465–2.135)	<0.001
Empty-stadium proxy	46	276	1.849 (1.337–2.558)	<0.001	1.841 (1.144–2.965)	0.012
Barcelona home	261	1,566	1.409 (1.223–1.623)	<0.001	1.825 (1.397–2.383)	<0.001
Barcelona away	258	1,548	1.565 (1.379–1.776)	<0.001	1.702 (1.340–2.162)	<0.001
Complete referee field	414	2,484	1.375 (1.236–1.530)	<0.001	1.886 (1.560–2.281)	<0.001
2012/13 onward	306	1,836	1.488 (1.306–1.696)	<0.001	1.547 (1.213–1.974)	<0.001

Sensitivity models followed the H1 specification with match-clustered robust standard errors. When a stratifying variable was constant within a subgroup, it was omitted. The complete-referee-field analysis was restricted to 414 matches with non-missing referee information; referee identity was not included in the model. Subgroup estimates assess directional consistency and robustness; differences in point estimates alone do not establish between-group effects.

## 4 Discussion

This study included 519 Barcelona La Liga matches across 17 seasons in which Messi had a recorded playing position. After adjustment for recorded foul intensity, total card-event rates were higher at 75–90 min and in second-half stoppage time than at 0–75 min. The Bad Behaviour share was larger in stoppage time, whereas the stoppage-time IRR for Foul Committed-linked cards did not indicate an increase. Home-trailing status at window onset was not clearly associated with more subsequent away-team cards, and there was no clear evidence of era differences in temporal associations. These observations describe temporal patterns in the density and composition of disciplinary events within the selected sample; they do not demonstrate referee bias.

Interpretation of H1 requires distinguishing conditional temporal associations, event-source composition, and differences in source-specific effects. Total-card IRRs were 1.487 at 75–90 min and 1.766 at >90 min; the >90-min IRR for Foul Committed-linked cards was 0.967. The Bad Behaviour share increased from 16.11% to 43.09%. These findings are consistent with a change in card-event composition, but significance in one model and non-significance in another do not establish that the Bad Behaviour temporal association is stronger than the Foul Committed-linked association. Without a direct Bad Behaviour model or an event-source × period interaction, its independent contribution cannot be quantified. The Bad Behaviour label also does not reliably distinguish dissent, delay, or other specific triggers.

Experimental and cognitive research provides a plausible psychological context for temporal changes in officiating. Referee decisions may be associated with self-control demands, analytical versus intuitive processing, anxiety, and physical fitness (Samuel et al., 2018; Castillo-Rodríguez et al., 2023; Wang et al., 2024; Zhang et al., 2024). Wang et al. (2024), for example, reported differences in accuracy across decision-making modes and showed that confidence need not align with accuracy. Nevertheless, the present study did not measure referee fatigue, anxiety, attentional allocation, confidence, or decision correctness, and it did not include independent blinded video review. Match time therefore cannot be treated as a direct proxy for cognitive-resource depletion, and the temporal coefficients should not be interpreted as evidence for a specific psychological mechanism. The more defensible conclusion is that real-match event streams show an observed shift in the composition of late disciplinary events; the psychological processes underlying that shift require studies with direct psychological or experimental measures.

The lack of support for H2 does not imply that crowd-based social pressure is absent. The present outcome was the card-event rate, the sample was restricted to Barcelona matches featuring Messi, and exposure was defined by score state at window onset. Earlier research has used added-time duration, full-match card differences, or broader league samples (Garicano et al., 2005; Nevill et al., 2002; Pettersson-Lidbom and Priks, 2010; Bryson et al., 2021; Reade et al., 2022; Lago-Peñas and Gómez-López, 2016). Because score state remained fixed within each window even if a goal occurred, these results cannot be interpreted as instantaneous card-event associations during the actual time the home team was trailing.

The lower home-team card-event rate in >90-min windows that began with the home team trailing (IRR = 0.589, *p* = 0.042; Table 4) requires particular caution. The home-away effect difference was not significant (interaction *p* = 0.493), stoppage-time exposure was short and events were sparse, and multiple window and subgroup comparisons were not adjusted. This isolated estimate should therefore be treated as exploratory rather than as evidence for H2 or a specific directional bias.

H3 was an exploratory era comparison. League-level research on VAR and home advantage or disciplinary differences has produced mixed findings (Dufner et al., 2023; Gasparetto and Loktionov, 2023; Işın and Yi, 2024). Here, the 75–90-min × era interaction had *p* = 0.072 and the >90-min × era interaction had *p* = 0.297. Their confidence intervals and the smaller later-era sample leave uncertainty about possible changes. The 2018/19 cut-off also captures contemporaneous changes in refereeing guidance, laws, team composition, and pandemic conditions, preventing separation of the effects of actual VAR interventions.

Using period-specific timestamps, score state at window onset, and separate card-event sources reduces misclassification due to rounded minutes, retrospective assignment of final scores, and mixed outcome definitions. Important limitations remain: the final recorded event may precede the end of the match and underestimate stoppage-time exposure; score state at window onset does not represent actual trailing time within the window; and recorded fouls depend on referee recognition and data recording. Adjustment for recorded foul intensity yields a conditional comparison, cannot remove shared measurement processes, and does not replace estimation of the overall temporal association without foul adjustment.

From an applied sport-psychology perspective, the results are more useful for match-management and training design than for ranking referee performance. The increased proportion of Bad Behaviour cards in stoppage time suggests value in training scenarios that combine high-pressure communication, dissent management, recognition of time-wasting behavior, and attentional control. Video-based feedback training has previously been used to improve intuitive decision-making skills in soccer referees (Schweizer et al., 2011). Evaluation of such training should combine decision accuracy, response time, confidence calibration, and match-control outcomes rather than equating more or fewer cards with better performance.

Future work should test preregistered temporal and score-state hypotheses in complete league or multi-league samples and, where possible, link event data to actual VAR interventions, verified attendance, referee identity, match importance, and more granular reasons for cards. Independent blinded video adjudication would allow event frequency to be separated from decision correctness. Statistically, cross-classified models for referees and matches could better represent the data-generating structure, sparse counts in short stoppage-time exposures could be modeled explicitly, and false-discovery control could be applied to multi-window comparisons. Combining event streams with video-based correctness labels and direct psychological or physical measures of referees would be necessary to distinguish among escalating player behavior, changes in match-management strategy, and changes in referee judgment.

## 5 Limitations

First, the sample was restricted to Barcelona matches featuring Messi and is subject to club-, player-, and open-data coverage selection. Second, recorded fouls and cards are post-decision events rather than independent behavioral gold standards. A temporal model without adjustment for same-period recorded foul intensity was not fitted, preventing direct comparison of overall and conditional associations. Third, first-half stoppage time was not examined descriptively or in a sensitivity analysis, so the findings cannot establish whether the observed pattern is specific to the end of the match or common to stoppage-time periods. Second-half stoppage-time exposure was also proxied by the final recorded event timestamp. Fourth, Bad Behaviour records lack granular reasons, and neither a direct model of those cards nor a formal event-source × period interaction was fitted; source-specific temporal differences are therefore unconfirmed. Fifth, score-state exposure was fixed at window onset, so within-window goals may cause state misclassification. Sixth, the era indicator does not identify actual VAR interventions and overlaps with contemporaneous changes. Seventh, empty-stadium status was proxied by dates without match-level attendance verification. Eighth, referee information was incomplete and referee identity was not modeled. Ninth, window-specific and subgroup comparisons were not adjusted for multiple testing. Tenth, there was no independent video-based adjudication or measurement of referee psychological or physical state, precluding direct tests of bias or cognitive mechanisms.

## 6 Conclusion

Across 519 Barcelona La Liga matches featuring Messi, total card-event rates were higher at 75–90 min and in second-half stoppage time than at 0–75 min after adjustment for recorded foul intensity. The Bad Behaviour share was larger in stoppage time, but the analyses did not formally establish different temporal associations by event source. Home-trailing status at window onset was not significantly associated with more subsequent away-team cards, and the era comparison provided no clear evidence of changed temporal associations from 2018/19 onward. These findings describe disciplinary-event patterns in the selected sample and do not establish changes in decision accuracy or referee bias.

## Data Availability

The original contributions presented in the study are included in the article/Supplementary material, further inquiries can be directed to the corresponding author.
