# Supplementary material for: Late-match card-event rates and score-state associations in Barcelona La Liga matches featuring Lionel Messi: a retrospective analysis of StatsBomb open event data
Source: Front Psychol. 2026 Oct 28;17:1992963. doi: 10.3389/fpsyg.2026.1992963 (PMC13751456; doi:10.3389/fpsyg.2026.1992963)
Supplement: Supplementary file 1 [file Table_1.docx]

# Supplementary Table S1

Data files, key fields, and verification results (n = 519).

| **Field** | **Unit** | **Expected** | **Available** | **Missing, %** | **Use/handling** |
| --- | --- | --- | --- | --- | --- |
| Match metadata | Match | 519 | 519 | 0.00 | Defines included match list |
| Event stream | Match | 519 | 519 | 0.00 | Reconstructs periods, score state, fouls, and cards |
| Lineup | Match | 519 | 519 | 0.00 | Confirms Messi had a recorded playing position |
| Referee field | Match | 519 | 414 | 20.23 | Excluded from primary model; used in complete-record sensitivity analysis |
| Stadium field | Match | 519 | 519 | 0.00 | Completeness check only |
| Attendance | Match | 519 | 0 | 100.00 | Date-based empty-stadium proxy |
| Actual VAR intervention | Match | 519 | 0 | 100.00 | 2018/19-or-later era proxy only |
| Final-score verification | Match | 519 | 519 | 0.00 | Event reconstruction matched match metadata |

Note. Missingness is calculated over 519 matches. The empty-stadium and era indicators are researcher-constructed proxies and do not substitute for observed match-level attendance or individual VAR intervention records. The complete-referee-field sensitivity analysis restricts the sample to non-missing records and does not include referee identity in the model.
